# Identification and Management of Fusobacterium Nucleatum Liver Abscess and Bacteremia in a Young Healthy Man

**DOI:** 10.7759/cureus.12303

**Published:** 2020-12-26

**Authors:** Shiliang A Cao, Sherifat Hinchey

**Affiliations:** 1 Anesthesiology, Harvard Medical School, Boston, USA; 2 Anesthesiology, Massachusetts General Hospital, Boston, USA; 3 Internal Medicine, Signature Healthcare Brockton Hospital, Brockton, USA

**Keywords:** fusobacterium, liver abscess, bacteremia

## Abstract

A 21-year-old previously healthy young man was admitted with five days of fever, persistent cough, worsening shortness of breath, and vomiting. On presentation, laboratory evaluation revealed extremely elevated procalcitonin and leukopenia followed by leukocytosis. The patient was started on empiric antibiotics. Further diagnostic evaluation after initiation of antibiotics included a computed tomography scan, which revealed a large hepatic abscess. Blood cultures obtained on admission grew *Fusobacterium nucleatum*; fluid obtained from the hepatic abscess also grew *F. nucleatum*. The patient’s antibiotic regimen was narrowed for specific coverage of *F. nucleatum*. The liver abscess was drained several times via image-guided percutaneous abscess drainage, with eventual resolution of the abscess. Patient received a prolonged course of intravenous antibiotics and, once stabilized, was discharged on two weeks of Augmentin. Here, we present a rare case of hepatic abscess and bacteremia due to *F. nucleatum* in a previously healthy young man with good oral hygiene. With this case, we aim to demonstrate the following: (1) the acute onset and rapid disease progression of *F. nucleatum* bacteremia and liver abscess; (2) how extreme procalcitonin elevation may serve to be a clinically useful early marker of *F. nucleatum* infection; and (3) the importance of early diagnosis, treatment, and definitive abscess drainage of *F. nucleatum* bacteremia and liver abscess.

## Introduction

Pyogenic liver abscesses are collections of pus in the liver due to a bacterial infection of the liver parenchyma. Pyogenic liver abscesses are rare, with an incidence rate of around 2.3 cases per 100, 000 patients [1]. Commonly involved organisms include enteric bacterial organisms such as *Escherichia coli *[2]. Most cases of hepatic abscesses in existing literature have been described as polymicrobial and have been found to be due to biliary tract disease, intra-abdominal infections, or trauma [3].

We describe *Fusobacterium nucleatum* as a rare cause of mono-microbial pyogenic liver abscess. *F. nucleatum* is an anaerobic gram-negative bacilli that is commonly found in the dental plaque of many primates [4]. Fusobacterium bacteremia is rare, with an incidence rate around 0.34 to 0.99 per 100, 000 patients [5,6]. Typically, systemic *F. nucleatum* infections occur in immunocompromised individuals or originate from the oral cavity.

Here, we present a case of *F. nucleatum* bacteremia and liver abscess in an immunocompetent man with reported good oral hygiene. Notably, our patient presented with a mono-microbial pyogenic liver abscess and bacteremia with *F. nucleatum* as the causative organism. While the etiology of this patient’s bacteremia and liver abscess is atypical, we believe it is important for physicians to be aware of liver abscess as a cause of fever and vomiting. Timely diagnosis and treatment are crucial, as pyogenic liver abscesses and bacteremia can be fatal if left untreated. We found procalcitonin to be an useful early marker of infection and appropriate treatment in this case of *F. nucleatum* liver abscess and bacteremia. Further, *F. nucleatum* as a cause of pyogenic liver abscess and bacteremia may warrant a place in early differential diagnosis and empiric antibiotic coverage.

## Case presentation

A 21-year-old nonverbal male with a past medical history of developmental delay, deafness, seizure disorder, and a remote history of childhood meningitis resulting in deafness, with a BMI of 35.3 kg/m2, was admitted with five days of fever, persistent cough, worsening shortness of breath, and an episode of vomiting the night prior to admission. The patient had been evaluated two days earlier for similar symptoms of fever, loss of appetite, and an episode of vomiting the night prior to presentation; at the time, he tested negative for influenza and streptococcus and was discharged home with presumed gastroenteritis. He re-presented two days later with persistent symptoms. The patient was nonverbal, but the mother of the patient reported that the patient may also be experiencing some abdominal pain. On presentation, the patient was febrile to 102 degree Fahrenheit, tachycardic to a rate of 145 beats per minute, and tachypneic to 60 breaths per minute. Blood pressure was 108/59 mmHg, and the patient was saturating 99% on room air. On physical examination, patient appeared to be in respiratory distress. His head and neck exam was notable for dry mucus membranes. His pulmonary exam revealed clear breath sounds. His cardiac exam revealed tachycardia, but normal rhythm. His abdominal exam revealed soft abdomen without rigidity or rebound; the patient did not show signs of pain to palpation of the abdomen. There was no lower extremity edema. Skin was intact, with no rashes or lesions. A presenting electrocardiogram revealed sinus tachycardia with incomplete right bundle branch block, but no acute ischemic changes. Chest x-ray showed clear lungs, with no abnormalities. Laboratory evaluation revealed leukopenia to 2 x 103/µL, procalcitonin of 33.26 ng/mL, and lactic acid of 7.0 mmol/L. His blood gas was notable for acidosis to a pH of 7.25 and pCO2 (partial pressure of carbon dioxide) elevation to 53 mmHg. His metabolic panel revealed no electrolyte abnormalities. Urinalysis revealed no leukocyte esterase, no white blood cells, and no bacteria. The patient tested negative for hepatitis, HIV, blood parasites, and Entamoeba histolytica. Anaerobic and aerobic blood cultures were obtained.

**Figure 1:**
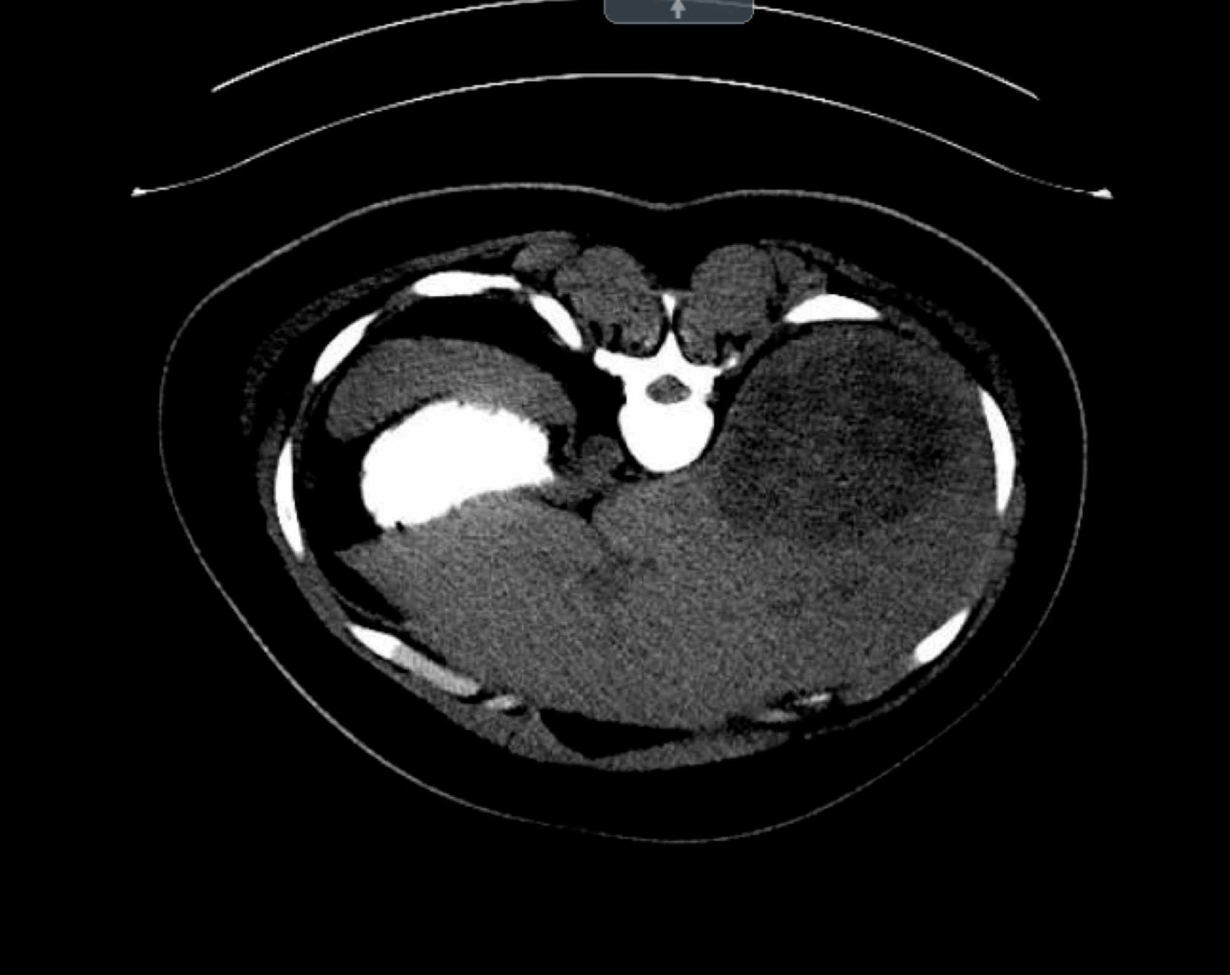
Computed tomography scan of the liver An abdominal computed tomography scan revealed a large right hepatic hypodensity measuring 10 cm x 8.5 cm. There was no intra- or extra-hepatic biliary ductal dilation. The gallbladder was unremarkable.

**Figure 2:**
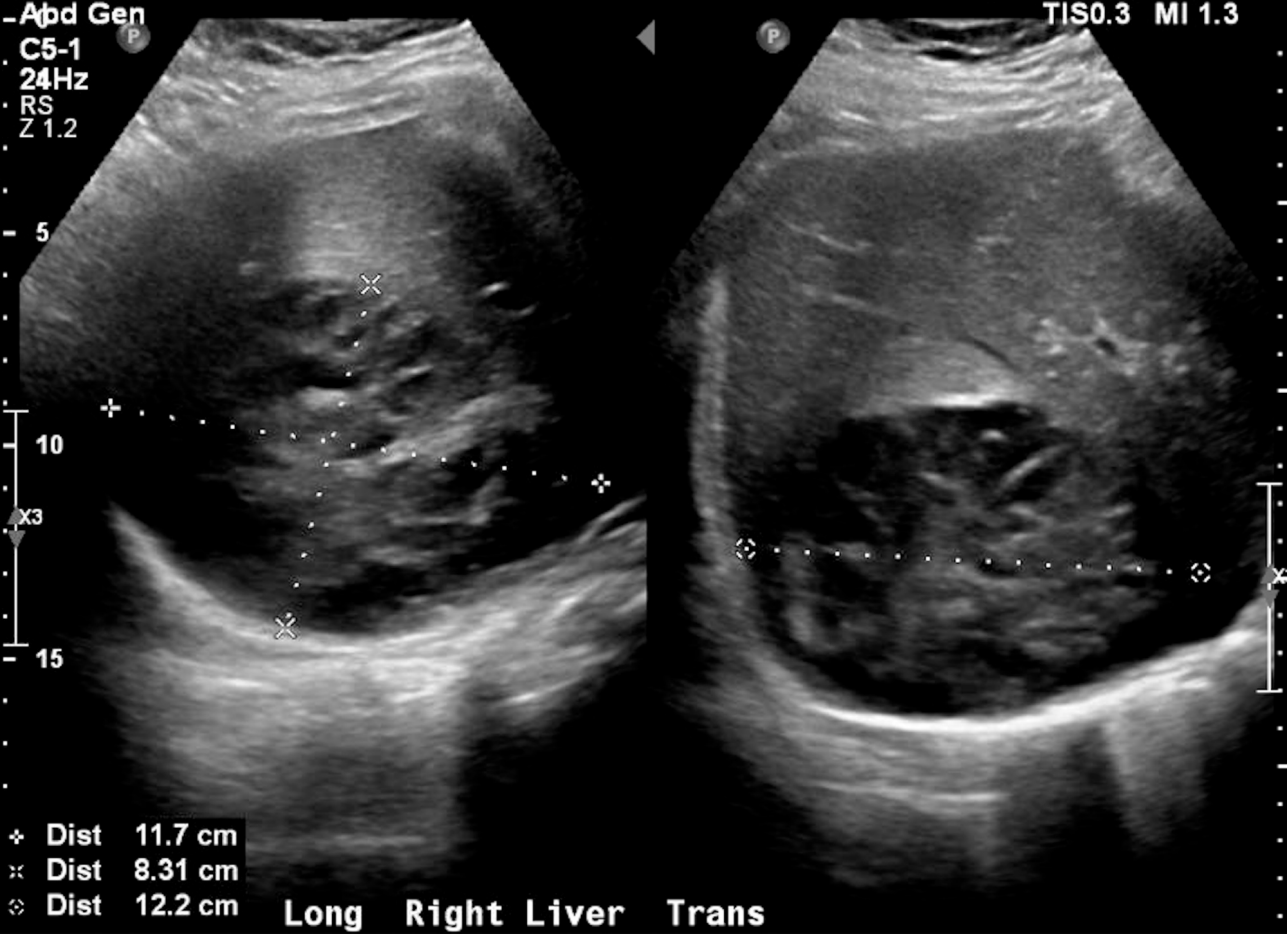
Ultrasound of the liver A right upper quadrant ultrasound revealed a large right hepatic complex solid and cystic mass measuring 11.7 cm x 12.2 cm x 8.3 cm, consistent with computed tomography findings.

An abdominal computed tomography scan was then performed, which revealed a large right hepatic hypodensity measuring 10 cm x 8.5 cm; otherwise, there was no intra- or extra-hepatic biliary ductal dilation, and the gallbladder was unremarkable (Figure 1). A right upper quadrant ultrasound was performed, which revealed a large right hepatic complex solid and cystic mass within measuring 11.7 cm x 12.2 cm x 8.3 cm, consistent with computed tomography findings (Figure 2). Together, the imaging findings were concerning for the possibility of malignancy versus abscess.

The patient was aggressively resuscitated with IV fluids and broad spectrum antibiotics, vancomycin and ceftriaxone. Given that the patient was febrile, tachypneic to the 60 breaths per minute, and tachycardic to 145 beats per minute with borderline blood pressure 108/59 mmHg, the patient was transferred to the intensive care unit for management of severe sepsis. The patient was placed on intermittent non-invasive positive pressure ventilation, with rapid improvement in his acidosis and respiratory status. In the ensuing days, the patient’s blood cultures grew *F. nucleatum*. His antibiotic coverage was transitioned to piperacillin-tazobactam and metronidazole. In evaluating the source of the bacteremia, the patient underwent a maxillary and facial computerized tomography scan, which showed no evidence of dental abscesses or other potential sources of seeding infection. While on antibiotic regimen, the patient underwent imaging-guided percutaneous drainage of the hepatic abscess. Specimen from the percutaneous drainage was cultured and grew *F. nucleatum*. The specimen showed acute suppurative inflammation but no evidence of malignant tumor cells. Repeat computed tomography scan revealed persistent loculated hepatic fluid collection, and percutaneous drainage was performed again to try and adequately drain the abscess. Two percutaneous drains were left in place. Specimen from the percutaneous drainage was sent for gram stain and culture, which grew *F. nucleatum*, similar to the blood culture. The patient was stabilized after percutaneous drainage and treatment with 22 days of antibiotics. The patient was then transferred to the hepatobiliary service of a tertiary care center for surgical evaluation for possible surgical debridement of the hepatic abscess. At the tertiary care center, the patient was continued on piperacillin-tazobactam. The patient underwent another interventional radiology hepatic abscess drainage of both percutaneous drains, with scant amount of pleural fluid aspirated. In the ensuing week, the patient remained stable clinically, and there was no output from either percutaneous drain. A computed tomography scan revealed improvement in the right lobe of the liver and no new undrained liver abscesses. The percutaneous drains were removed by interventional radiology. The patient was discharged to home in improved medical condition with two weeks of oral Augmentin.

**Figure 3:**
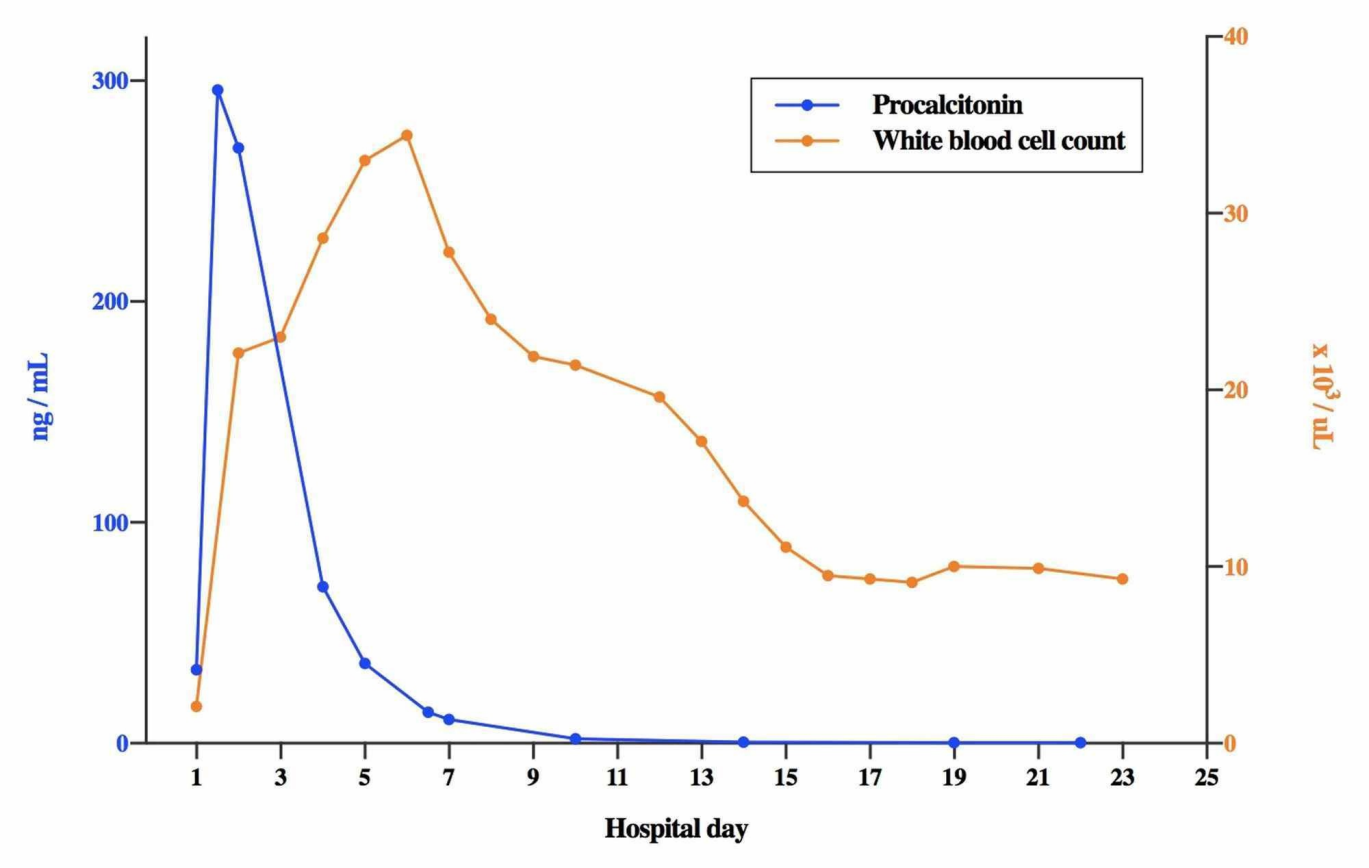
Procalcitonin level and white blood cell count over time Our patient presented with a procalcitonin of 33.26 ng/mL, which rapidly rose and peaked at 295.74 ng/mL in a matter of hours before slowly downtrending after initiation of fluids, antibiotics, and liver abscess drainage. The changes in procalcitonin level preceded similar changes in the patient’s white blood cell count. The patient’s white blood cell count was 2.1 x 103/µL on arrival, rose to 22.1 x 103/µL on hospital day two, peaked at 34.4 x 103/µL on hospital day six before slowly downtrending to normal levels by the time of discharge.

Of note, the patient’s procalcitonin level peaked at 295.74 ng/mL on the first day of his hospital admission and downtrended from there with initiation of broad spectrum antibiotics and percutaneous drainage (Figure 3). The patient was leukopenic with a white blood cell count of 2.1 x 103/µL on arrival; however, the patient’s white blood cell count rose quickly to 22.1 x 103/µL on hospital day 2, peaking at 34.4 x 103/µL on hospital day six before slowly downtrending to normal levels by the time of discharge (Figure 3). The patient had elevated aspartate aminotransferase (AST) (206 U/L), alanine aminotransferase (ALT) (peak 138 U/L), and alkaline phosphatase (160 U/L) on the day of arrival; however, this quickly resolved after the first day, with no abnormalities in liver enzymes thereafter. The patient had lipase elevated to 144 U/L on arrival; this peaked at 225 U/L and quickly downtrended to normal levels. The patient had normal bilirubin throughout his stay. The patient also had a mild elevation in creatinine on the day of arrival to 1.8 mg/dL, which slowly resolved throughout his stay.

## Discussion

Fusobacterium

Fusobacterium is a small non-motile, anaerobic, gram-negative spindle-shaped rod. Fusus means spindle, and bacterion means small rod [7]. *F. nucleatum* is part of normal oral flora and is typically associated with infections of the head and neck, such as gingival and periodontal disease [8].

*Fusobacterium nucleatum* bacteremia

Anaerobic bacteremia is rare, occurring in less than 1% of all positive blood cultures [9]. Furthermore, Fusobacterium species account for less than 1% of anaerobic bacteremias and less than 0.001% of all bacteremias [5]. *Fusobacterium necrophorum* bacteremia tends to occur in younger patients without underlying comorbidities, while *F. nucleatum* bacteremia, as seen in our case, tends to occur in older populations with other issues such as underlying malignancy [5]. Our case is unique in that the *F. nucleatum* bacteremia occurred in a young, previously healthy 21-year-old man with no underlying issues such as malignancy and with no reported gingival or periodontal disease. *F. nucleatum* bacteremia has been reported to have a high mortality rate, with some hospitals reporting a 30-day mortality rate of up to 47.4% [10]. Fortunately, in our case, the patient recovered following a prolonged course of antibiotics and sequential percutaneous drainage of his hepatic abscess.

Liver abscesses

There are two main types of liver abscesses: pyogenic liver abscess and amoebic abscess. Pyogenic liver abscess is a hepatic lesion that typically contains blood or pus. The annual incidence of pyogenic liver abscess is around 2.3 cases per 100,000 per year [2]. The most common organisms found in pyogenic liver abscess include *Klebsiella pneumonia*, *Streptococcus melleri*, and *E. coli* [2]. Only one other case that we know of has reported pyogenic liver abscess caused by *F. nucleatum* in an otherwise young and healthy immunocompetent patient [11]. However, in the case reported by Ahmed et al., the patient was not noted to have bacteremia. We report what we know to be the second case of *F. nucleatum* pyogenic hepatic abscess in a young immunocompetent patient, and the only reported case of *F. nucleatum* causing both pyogenic hepatic abscess and bacteremia in a young immunocompetent patient.

Procalcitonin

Procalcitonin, a precursor for calcitonin, is synthesized by various cells during sepsis [12]. It has been shown to correlate with sepsis and infections, particularly severe bacterial infections [13]. Several studies have commented on the usefulness of procalcitonin as a sensitive and specific marker of bacterial infections [14-16]. Few studies have reported procalcitonin levels in relation to *F. nucleatum *infections. A study by Leli et al. showed that *F. nucleatum* bloodstream infections correlated to a median procalcitonin level of about 0.38 ng/mL [17]. One case report described a procalcitonin level elevated to 294 ng/mL in a patient with *F. necrophorum* pharyngotonsillitis, Lemierre’s syndrome, and bacteremia [18]. This case report describes a rare case of extreme procalcitonin elevation to over 200 ng/mL in the setting of *F. nucleatum* liver abscess and bacteremia. Our patient presented with a procalcitonin of 33.26 ng/mL, which rapidly rose and peaked at 295.74 ng/mL in a matter of hours before slowly downtrending after initiation of fluids, antibiotics, and liver abscess drainage. Interestingly, the changes in procalcitonin level preceded similar changes in the patient’s white blood cell count. The delayed increase and decrease in white blood cell count in the first few days of the patient’s hospitalization appeared to be a delayed reflection of the rise and decline in procalcitonin levels, which promptly responded to antibiotic therapy on the first day of the patient’s hospital course (Figure 3). Xiao et. al found in their clinical trial that procalcitonin is a more reliable predictor than white blood cell count in detecting infections following radical gastrectomy for gastric cancer [19]. Another group found that procalcitonin alone, or when combined with white blood cell count, was the best diagnostic and prognostic biomarker of infection or sepsis in patients presenting to the emergency department [20]. Consistent with these studies, we observed an early change in procalcitonin levels that preceded a corresponding change in white blood cell count. We propose that procalcitonin may be an early useful diagnostic marker of *F. nucleatum* liver abscess and/or bacteremia, one that may even precede changes in a patient’s white blood cell count.

## Conclusions

This article highlights a case of mono-microbial pyogenic liver abscess and bacteremia caused by *F. nucleatum*. A 21-year-old man presented with fevers, worsening shortness of breath, and possible abdominal pain and was found to have bacteremia and a liver abscess measuring 11.7 cm x 12.2 cm x 8.3 cm, both eventually speciating *F. nucleatum*. He was started on vancomycin and ceftriaxone, then switched to piperacillin-tazobactam and metronidazole, and then narrowed to piperacillin-tazobactam only. The abscess was drained several times via image-guided percutaneous abscess drainage, with eventual resolution of the abscess. The patient was discharged in two weeks of amoxicillin-clavulanic acid. Lab abnormalities include brief transaminitis and elevated lipase, extreme procalcitonin elevation, and leukopenia followed by rapid rise and eventual decline in white blood cell count, which paralleled procalcitonin levels in a delayed manner. Despite the rarity of these cases, pyogenic liver abscess should be considered as a differential diagnosis in patients with fever and vomiting. Procalcitonin should be considered as a potentially useful biomarker that may be able to identify *F. nucleatum* infections early, perhaps even before a rise in white blood cell count. Reassuringly, our case suggests that patients with *F. nucleatum* liver abscess and bacteremia may recover with appropriate antibiotics and adequate percutaneous abscess drainage. Further research is needed to elucidate the pathophysiology of *F. nucleatum* causing pyogenic liver abscess and bacteremia in a young and previously healthy male.
